# Safety and Efficacy of the FRED Jr Flow Re-Direction Endoluminal Device for Intracranial Aneurysms: Retrospective Multicenter Experience With Emphasis on Midterm Results

**DOI:** 10.3389/fneur.2021.722183

**Published:** 2021-10-01

**Authors:** Jessica Jesser, Nilüfer D. Alberalar, Osman Kizilkilic, Isil Saatci, Feyyaz Baltacioglu, Enes Özlük, Monika Killer-Oberpfalzer, Dominik F. Vollherbst, Civan Islak, Saruhan H. Cekirge, Martin Bendszus, Markus Möhlenbruch, Naci Koçer

**Affiliations:** ^1^Department of Neuroradiology, Heidelberg University Hospital, Heidelberg, Germany; ^2^Department of Diagnostic and Interventional Radiology, University Hospital of Leipzig, Leipzig, Germany; ^3^Department of Neuroradiology, Cerrahpasa Medical Faculty, Istanbul, Turkey; ^4^Interventional Neuroradiology Section, Koru and Bayindir, Private Hospitals, Ankara, Turkey; ^5^Department of Radiology, School of Medicine, Marmara University, Istanbul, Turkey; ^6^Department of Radiology, Acibadem Atakent Hospital, Istanbul, Turkey; ^7^Department of Radiology, Paracelsus University, Salzburg, Austria

**Keywords:** flow diversion, cerebral small vessels, FRED junior, low-profile flow diverter, intracranial aneurysm

## Abstract

**Background and Purpose:** Flow diversion is increasingly used as an endovascular treatment for intracranial aneurysms. In this retrospective multicenter study, we analyzed the safety and efficacy of the treatment of intracranial, unruptured, or previously treated but recanalized aneurysms using Flow Re-Direction Endoluminal Device (FRED) Jr with emphasis on midterm results.

**Materials and Methods:** Clinical and radiological records of 150 patients harboring 159 aneurysms treated with FRED Jr at six centers between October 2014 and February 2020 were reviewed and consecutively included. Clinical outcome was measured by using the modified Rankin Scale (mRS). Anatomical results were assessed according to the O'Kelly-Marotta (OKM) scale and the Cekirge-Saatci Classification (CSC) scale.

**Results:** The overall complication rate was 24/159 (16%). Thrombotic-ischemic events occurred in 18/159 treatments (11%). These resulted in long-term neurological sequelae in two patients (1%) with worsening from pre-treatment mRS 0–2 and mRS 4 after treatment. Complete or near-complete occlusion of the treated aneurysm according to the OKM scale was reached in 54% (85/158) at 6-month, in 68% (90/133) at 1-year, and in 83% (77/93) at 2-year follow-up, respectively. The rates of narrowing or occlusion of a vessel branch originating from the treated aneurysm according to the CSC scale were 11% (12/108) at 6-month, 20% (17/87) at 1-year, and 23% (13/57) at 2-year follow-up, respectively, with all cases being asymptomatic.

**Conclusions:** In this retrospective multicenter study, FRED Jr was safe and effective in the midterm occlusion of cerebral aneurysms. Most importantly, it was associated with a high rate of good clinical outcome.

## Introduction

The treatment of intracranial aneurysms of complex morphology, such as aneurysms with a wide neck, irregular shape (e.g., daughter sac aneurysms), or aneurysms with incorporated branching vessels, may be unfavorable or even unsuitable for traditional endovascular or microsurgical treatments (1, 2). Also, recanalized aneurysms after endovascular or microsurgical treatment often show characteristics of complex morphology, limiting the options for successful retreatment (3, 4). In these cases, flow diversion stent treatment is an increasingly used alternative (1). The densely braided mesh of a flow diversion stent delivered at the aneurysm base decreases the blood flow into the aneurysm and induces thrombus formation within the aneurysm sac while maintaining adequate blood flow through the parent artery and the covered vessel branches (5). The development of smaller flow diverters like Flow Re-Direction Endoluminal Device Jr (FRED Jr; MicroVention, Tustin, California), which can be delivered into small-caliber (<3 mm diameter) vessels, enabled the expansion of indications for flow diversion stent treatment in smaller parent vessels (2, 6). However, there is only a limited number of studies evaluating flow diverter devices specifically developed for smaller arteries (2, 7).

In this study, we evaluate the applicability of flow diverter stent treatment using the FRED Jr for unruptured or previously treated but recanalized intracranial aneurysms in smaller vessels and examine the device's safety and efficacy.

## Methods

### Patient and Case Selection

This retrospective study was approved by the local ethics committees. Treatment decisions were made by a multidisciplinary team (vascular neurosurgeons and interventional neuroradiologists) on a case-by-case basis. Clinical and radiological records of all patients with intracranial, unruptured, or recanalized aneurysms treated with FRED Jr at six centers between October 2014 and February 2020 were reviewed and consecutively included. Midterm follow-ups of these patients were added. Patients' demographics were assessed, including age, sex, clinical presentation, and modified Rankin Scale (mRS).

Regarding the treated aneurysms, we evaluated the type, size, location, vessel diameter, and existence of branching vessels from the aneurysm sac itself or from the parent artery adjacent to the aneurysm (distance of ≤ 2 mm to the aneurysm neck), which needed to be covered by the flow diverter.

### Antiplatelet and Anticoagulant Therapy

All patients were treated either with dual or mono antiplatelet therapy. Antiplatelet therapy used during each procedure is listed in the Supplementary Material. The dual therapy combined 100–300 mg of acetylsalicylic acid (ASA) with 75 mg of clopidogrel (loading dose 300 mg), starting no <5 days before the treatment. Platelet inhibition was tested prior to procedures. During all procedures at all centers, weight-adjusted heparinization with an activated clotting time >250 s was maintained. Depending on the case and the imaging results during the follow-up period, the initial antiplatelet therapy was maintained for a minimum of 6 months after the procedure followed by ASA monotherapy for at least 6 months or lifelong. At four centers, especially for patients younger than 65 years, an initial daily mono antiplatelet medication with prasugrel 10 mg (loading dose 40 mg) was maintained for 1 year and thereafter either discontinued or continued with reduced doses of prasugrel or ASA lifelong.

### Description of Technique

After determining the shape, size, and neck width of the aneurysm, a suitable flow diverter, fully covering the neck, and safety margins of at least 2 mm proximally and distally to the aneurysm, was chosen. To document the degree of expansion and vessel wall adherence of the flow diverter, a flat detector computed tomography angiography (FDCTA) was performed during and/or after the deployment. Additional coiling was performed in jailing technique in cases with high rupture risk or where complete occlusion with sole flow diverter treatment was deemed unlikely. Furthermore, data from retreatment procedures were collected.

### Clinical Evaluation

Clinical evaluation, including mRS, was documented by an experienced neurointerventionalist, neurologist, or neurosurgeon 1 day after the procedure, the following day, at discharge, and at follow-up examinations, respectively. Good clinical outcome at follow-up was defined either as mRS 0–2 at follow-up or an unchanged mRS prior unchanged to pre-treatment (8). Minor stroke was defined as NIHSS ≤ 3, and major stroke as NIHSS >3 (9).

### Anatomical Evaluation

Follow-up imaging, depending on the local standard of care, was performed either with contrast-enhanced MR-angiography, FDCTA, or DSA and interpreted by two senior neuroradiologists not involved in the treatment. The grade of aneurysm occlusion was rated according to the O'Kelly-Marotta (OKM) grading scale (10). The occlusion grade describes the degree of aneurysm filling after treatment (A = total, B = subtotal, C = entry remnant, and D = no filling). Adequate aneurysm occlusion was defined as OKM C and OKM D.

Furthermore, aneurysms were rated according to the Cekirge-Saatci Classification (CSC) (11). The CSC class describes not only the degree of aneurysm filling after treatment but also the patency of vessel branches arising from the treated aneurysm (Class 1 = complete occlusion, 1A: with full patency of the integrated branch, 1B: with the branch reduced in caliber, and 1C: with no antegrade filling of the branch; Class 2 = neck filling; Class 3 = incomplete occlusion with aneurysm filling; Class 4 = reserved for an immediate post-operative result based on end-of-treatment DSA, 4A: contrast stagnation, and 4B: without contrast stagnation; and Class 5 = stable remodeling with flow modification, i.e., filling in the neck region).

### Statistics

Patient and aneurysm characteristics as well as patients' clinical status (mRS) and aneurysm occlusion at follow-up were summarized using descriptive statistics and are presented as mean ± standard deviation (minimum–maximum) or as absolute number (relative frequency in percentage). For the descriptive statistics of clinical and anatomical results at different time points of follow-up, missing data were replaced by the last observation carried forward method. These data are included in the Supplementary Material.

Differences in variable distribution for the subgroups with complete vs. incomplete aneurysm occlusion at the 6-month follow-up were compared using a multivariate binary logistic regression with a *p*-value of 0.05 as the threshold for statistical significance. The same variables were tested for association with thrombotic-ischemic complications. These analyses were performed with SPSS Version 24 (IBM, Armonk, New York). Further details about the analyses can be found in the Supplementary Material.

We assessed the influence of vessel branches originating from aneurysms on occlusion rates for cases with 2-year follow-up examinations, by conducting a Kaplan–Meier analysis using GraphPad Prism 7 (San Diego, CA, USA).

## Results

### Patient and Aneurysm Characteristics

Overall, 150 patients (101 females and 49 males) and 159 aneurysms were treated between October 2014 and February 2020. In nine patients, two aneurysms in different locations were treated separately with one FRED Jr each. Mean patient age was 55 ± 12 years (15–81 years); 30/159 (19%) aneurysm cases were secondary treatments, due to residual aneurysm filling or regrowth after previous treatment with either clips (7 aneurysms), coils (19 aneurysms), woven endobridge (WEB) device (MicroVention, Aliso Viejo, USA) (1 aneurysm), or flow diverters other than FRED Jr (3 aneurysms). Aneurysms had a mean diameter of 6.7 ± 4.9 mm (1.0–36.0 mm) with a mean neck size of 4.0 ± 2.1 mm (1.1–14.5 mm). Proximal and distal mean diameters of the parent vessel were 2.4 ± 0.4 mm (1.4–3.6 mm) and 2.1 ± 0.4 mm (1.4–3.4 mm). One hundred eight (68%) of the treated aneurysms had a vessel arising either directly from the aneurysm or from the vicinity of the aneurysm sac, which was covered by the flow diverter. More characteristics and the locations of the treated aneurysms are given in Table 1 and Supplementary Material.

**Table 1:** Aneurysm characteristics.

	**% of total**
**Location**	
Distal ICA and PCom	2 (1%)
M1 of MCA	21 (13%)
Bifurcation of MCA	48 (30%)
Distal (to bifurcation) MCA	6 (4%)
A1 of ACA and ACom	34 (21%)
Distal (to ACom) ACA	32 (20%)
VA	3 (2%)
SUCA	1 (1%)
BA	1 (1%)
P1 of PCA	2 (1%)
Distal (to P1) PCA	9 (6%)
**Proximal vs. distal location**	
Proximal	112 (70%)
Distal (to MCA bifurcation, to ACom, to P1 of PCA)	47 (30%)
**Anterior vs. posterior circulation**	
Anterior	143 (90%)
Posterior	16 (10%)
**Aneurysm morphology**	
Blister	4 (3%)
Fusiform	12 (8%)
Dissecting	14 (9%)
Saccular	125 (79%)
Giant	4 (3%)
**Aneurysm size**	
Large (>1 cm)	27 (17%)
Small (<1 cm)	132 (83%)

*ACA, anterior cerebral artery; ACom, anterior communicating artery; BA, basilar artery; PCA, posterior cerebral artery; PCom, posterior communicating artery; SUCA, superior cerebellar artery; VA, vertebral artery*.

### Procedural Aspects and Complications

In all procedures, a single FRED Jr device could be successfully deployed across the aneurysm base to achieve complete coverage. Additional coiling was performed in six cases. In one case with a dysplastic bifurcation of the middle cerebral artery (MCA) with two associated aneurysms, a WEB device was placed into one of the aneurysms prior to deployment of a FRED Jr.

Overall, procedure-related complications were observed in 24/159 (16%) of treatments.

Technical complications occurred in 6/159 (3%) of the procedures. In five procedures, the chosen flow diverter was either too short or too long and had to be replaced. On one occasion, the distal portion of the FRED Jr opened insufficiently due to a stenosis of the parent artery distal to the aneurysm, and a balloon angioplasty was performed to achieve a satisfying result.

Thrombotic-ischemic complications occurred in 18/159 (11%) of the treatments. In 12/159 (7%) of the aneurysm treatments, in-stent thrombosis in the parent artery was seen during the procedure after deployment of the FRED Jr. In all of these cases, the administration of tirofiban could maintain or restore full vessel patency. Within the first 30 days after treatment, 9/159 (6%) patients suffered from periprocedural ischemic events. Seven out of these nine patients had minor strokes with transient symptoms. Two patients had major strokes with remaining clinical sequelae, worsening from an initial mRS of 0 to mRS 2 and mRS 4, respectively. Three of these complications, including the two cases of major strokes, were related to thrombosis in the parent vessel. In one case, 10 days after the treatment of an MCA bifurcation aneurysm, we noted a minimal stent deformation with distal caliber reduction, which could be described as “fishmouthing,” and the patient suffered from a transient mild paresis of the left hand.

In 1/159 (1%) procedures, a minor asymptomatic periprocedural subarachnoid hemorrhage occurred during flow diverter placement without clinical consequences.

No patient suffered from intraparenchymal hemorrhage after the treatment.

There was one patient (1/159; 1%) who developed a puncture site pseudoaneurysm and was successfully treated with surgery.

Regarding antiplatelet therapy, patients were medicated with ASA and clopidogrel in 69 treatments and with prasugrel in 90 treatments. Antiplatelet therapy with prasugrel was significantly predictive of fewer thrombotic-ischemic complications, according to a multivariate analysis (odds ratio 11.21, CI 2.93–42.86, *p*-value 0.0004; Table 2).

**Table 2:** Multivariate analysis of predicting factors for treatment-related complications (thrombotic or ischemic events) and of predicting factors for aneurysm occlusion conducted on 158 cases at 6-month follow-up.

	**Univariate**		**Multivariate**	
	**OR (95% CI)**	***p*-value**	**OR (95% CI)**	***p*-value**
**Independent variables for adequate aneurysm occlusion**				
Gender• (Female vs. male)	1.82	0.15	1.93	0.10
Age• (Continuous)	0.98	0.20		
Antiplatelet therapy• (ASA/clopi. vs. pras.)	0.77	0.50		
Shape• (Diss./Fusi. vs. Sacc.)	0.93	0.92		
Diameter• (Continuous)	0.93	0.24		
Neck size• (Continuous)	1.18	0.37		
Branch from aneurysm• (No vs. yes)	**4.80** (1.74–13.30)	**0.002**	**5.48** (2.05–14.66)	**0.001**
**Independent variables for thrombotic-ischemic complications**				
Gender (Female vs. male)	0.97	0.95		
Age (Continuous)	0.95 (0.91–0.99)	0.02	0.95 (0.91–0.99)	0.09
Antiplatelet therapy (ASA/clopi. vs. pras.)	**11.44** (2.88–45.40)	**0.001**	**11.21** (2.93–42.86)	**0.0004**
Shape (Diss./Fusi. vs. Sacc.)	2.19	0.38		
Diameter (Continuous)	1.08	0.42		
Neck size (Continuous)	0.87	0.40		
Branch from aneurysm (No vs. yes)	0.74	0.65		

*CI, confidence interval; Diss., dissecting; Dist., distal; Fusi., fusiform; OR, odds ratio; Prox., proximal; Sacc., saccular, vs., versus*.

*Values that reached statistical significance in the multivariate analysis are printed in bold*.

### Clinical and Anatomical Results Over Time

Clinical and anatomical results at 6 months were evaluated for 158/159 aneurysm treatments. One patient treated in 2018 was lost to follow-up due to Alzheimer's disease. Flow diverter deformation was seen in one case at 5-month follow-up, causing an asymptomatic stenosis of <50% of the distal parent vessel. One patient developed asymptomatic parenchymal lesions seen at the 4-month follow-up MRI, which regressed under steroid medication without further sequelae and could be described as non-ischemic cerebral enhancing (NICE) lesions (12). Two patients still suffered from sequelae of a major stroke with an mRS of 2 and 4, respectively. One aneurysm (also mentioned below in the *Patients Requiring Retreatment* section) required retreatment with parent artery occlusion due to aneurysm growth 3 months after the initial treatment and then suffered from a major ischemic stroke with worsening from mRS 0 to mRS 2. Thus, the rate of good clinical outcome at the 6-month follow-up was 99% (157/158). Aneurysm occlusions according to the OKM scale were OKM C 11% (18/158), OKM D 42% (67/158), and OKM C + D 54% (85/158), respectively. In 108/158 cases with a vessel branch arising from the aneurysm sac or from the parent vessel adjacent to the neck of the aneurysm, aneurysm occlusion rates were CSC 1A 23% (25/108), CSC 1B 6% (7/108), CSC 1C 5% (5/108), and CSC 5 6% (7/108), respectively. The narrowing (CSC 1B) or occlusion (CSC 1C) of a vessel arising from the aneurysm was asymptomatic in all 12 cases.

Clinical and anatomical results at 12 months were available for evaluation in 133/134 cases. One hundred thirty-four cases were treated before February 2019 and therefore eligible for the 12-month follow-up. As mentioned above, one patient was lost to follow-up due to Alzheimer's disease. No patient reported new neurological symptoms at the 12-month follow-up visit. Aneurysm occlusions according to the OKM scale were OKM C 15% (20/133), OKM D 53% (70/133), and OKM C + D 68% (90/133), respectively. In 87/133 cases with a vessel branch arising from the aneurysm, occlusion was CSC 1A 27% (23/87), CSC 1B 9% (8/87), CSC 1C 10% (9/87), and CSC 5 12% (10/87), respectively. The narrowing (CSC 1B) or occlusion (CSC 1C) of a vessel branch arising from the aneurysm was asymptomatic in all 17 cases.

Clinical and anatomical results at 24 months were evaluated in 93 cases, all of which were treated before February 2018 and were therefore eligible for the 24-month follow-up. Twelve of these cases were not yet screened at the 24-month follow-up. For these cases, results from the last observation were carried forward for the evaluation of the 24-month follow-up. No new neurological symptoms were reported at the 24-month follow-up. Aneurysm occlusions according to the OKM scale were OKM C 19% (18/93), OKM D 63% (59/93), and OKM C + D 83% (77/93), respectively. In 57/93 cases with a vessel branch arising from the aneurysm, occlusions were CSC 1A 37% (21/57), CSC 1B 11% (6/57), CSC 1C 12% (7/57), and CSC 5 16% (9/57), respectively.

**Figure 1:**
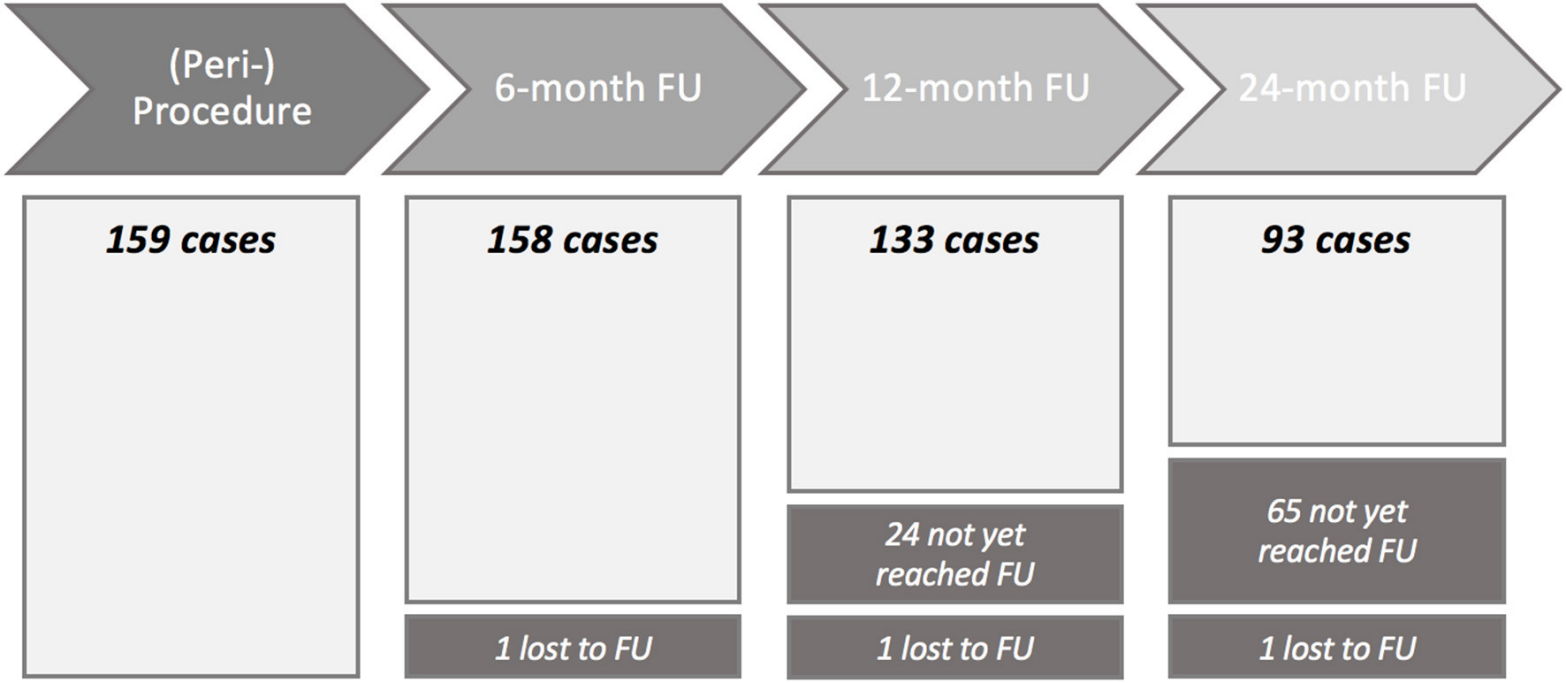
Flowchart of cases included into the analysis at each time point. FU, follow-up.

Figure 1 shows the number of cases included in the analysis over time.

### Predictors of Aneurysm Occlusion

Predictive factors for adequate aneurysm occlusion (OKM C + D) at the 6-month follow-up identified by a multivariate analysis are presented in Table 2. A branch arising from the aneurysm sac or its immediate vicinity was an unfavorable factor for adequate aneurysm occlusion (odds ratio 5.48, CI 2.05–14.66, *p*-value 0.001).

**Figure 2:**
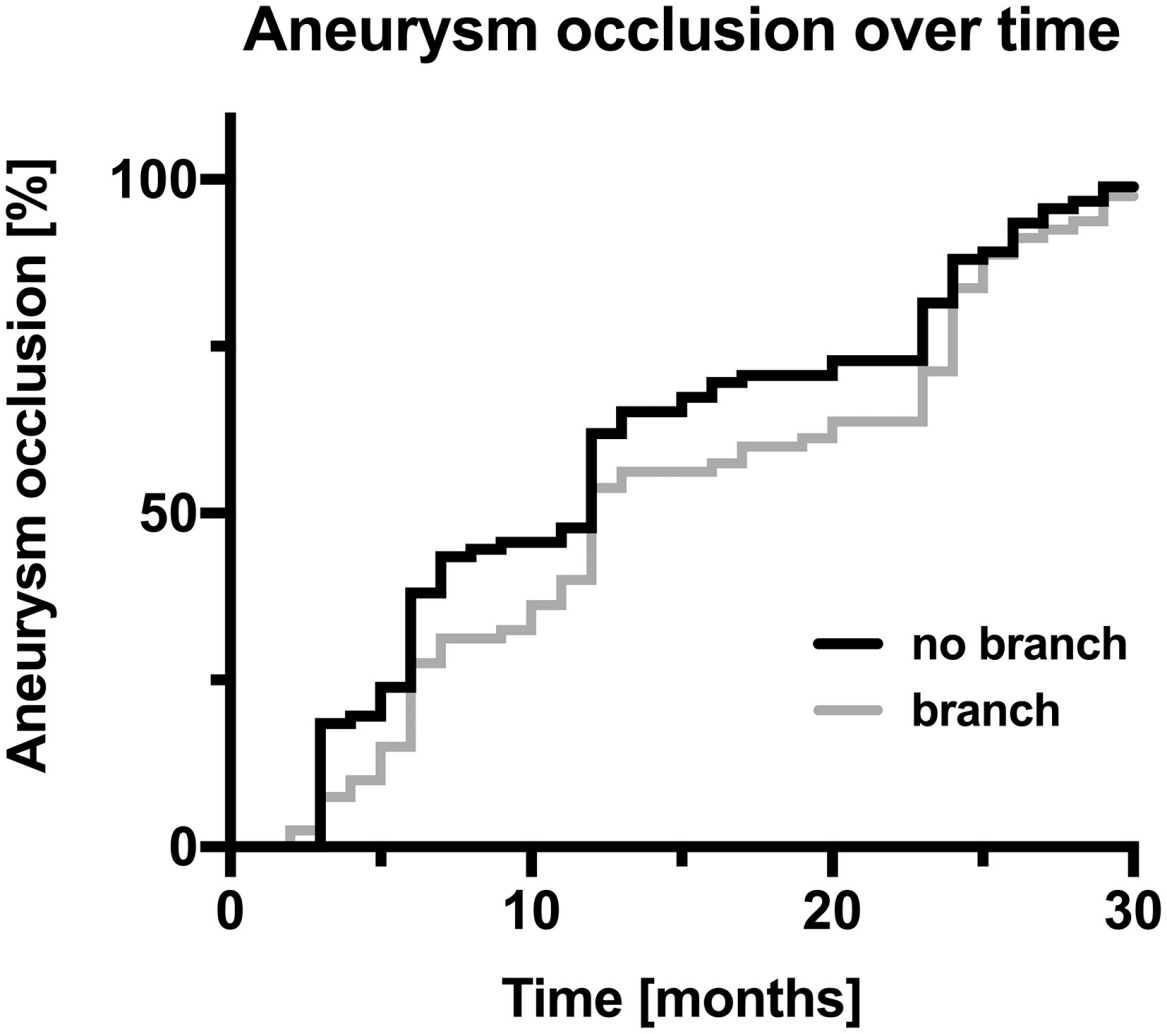
Kaplan–Meier curves of the percentage of aneurysm occlusion over time; cases comprising a branch originating from the aneurysm covered by the flow diverter are displayed in gray, and cases without a branch originating from the aneurysm are displayed in black.

When we compared occlusion rates in cases with vs. without incorporated vessel branches by a Kaplan–Meier analysis, we found a statistical trend (Gehan–Breslow–Wilcoxon test, chi square 2.905, *p*-value 0.088) for delayed occlusion of aneurysms with an incorporated vessel branch in the 93 cases available for analysis at 24-month follow-up (see Figure 2 for corresponding Kaplan–Meier curves).

### Patients Requiring Retreatment

In total, retreatment was necessary for 6/159 (4%) aneurysms. A dissecting aneurysm located at the P2-segment of the posterior cerebral artery (PCA) showed enlargement 3 months after flow diverter treatment, requiring coil occlusion of the parent artery, which resulted in an ischemic infarction distal to the occluded artery with worsening of the mRS from 0 to 2. In another dissecting PCA-P3 aneurysm, parent artery occlusion was performed 29 months after the initial treatment due to further growth of the aneurysm sac. Four more patients showed insufficient occlusion of the treated aneurysm upon follow-up, and an additional flow diverter was placed coaxially within the FRED Jr (for more details see the Supplementary Material).

## Discussion

In this retrospective multicenter study, the safety and efficacy of the FRED Jr for the treatment of intracranial aneurysms were investigated with emphasis on midterm results. The study represents a real-world scenario by including various types and locations of aneurysms treated in different international centers with variations in technical approaches, periprocedural management, and interventionalists' experience.

We demonstrated that aneurysm treatment with FRED Jr is overall safe in terms of neurological outcome. Although the overall complication rate of 16% is relatively high, it should be considered that many of the aneurysms featured a complex anatomy and also were associated with incorporated vessels and abruptly changing vessel diameter, which makes them more challenging to treat. In aneurysm cases associated with complex vessel anatomy settings, FRED Jr has the advantage of allowing the usage of a smaller 0.021-in microcatheter, which can facilitate vessel probing. Nevertheless, in 3% of treatments, technical complications were encountered mainly due to the inappropriate choice of the flow diverter size. We suppose that in smaller vessels with changing vessel diameters, the shortening of the flow diverter in its final location was more difficult to predict. Most complications were, in line with other studies, related to thrombotic events (7%) and could be mitigated effectively by the application of tirofiban with low rates of severe neurological complications (13). Despite a 6% rate of periprocedural ischemic events, there were only two patients who suffered from ischemia-related long-term neurological complications with mRS 2 and mRS 4, respectively, resulting in a good clinical outcome rate (mRS 0–2) of 99% in our study. Cagnazzo et al. reports an overall complication rate of 20% comprising 16% of thrombotic-ischemic events in a meta-analysis of flow diverter treatment in MCA aneurysms (14). This result is comparable to our results, when considering that 48% of the aneurysms in our study were located in the MCA. In our multivariate analysis, we found lower thrombotic-ischemic complications in patients treated with prasugrel for antiplatelet therapy. Prasugrel is a promising antiplatelet agent, showing less drug–drug interactions than clopidogrel or ticagrelor and a faster onset of action (15). Nevertheless, when interpreting this result, it should be noted that this medication was not consistently used in every study center, and other factors in periprocedural management may influence thrombotic-ischemic complications. Further studies randomized for antiplatelet treatment will be needed to uncover the true potential of variations in antiplatelet therapy.

When judging occlusion rates, one must consider that 68% of the aneurysms treated in our cohort incorporated vessel branches. Therefore, occlusion results cannot be easily compared to studies examining treatments in proximal vessel locations (16). The aforementioned meta-analysis by Cagnazzo et al. about flow diversion in MCA aneurysms reports that complete/near-complete occlusion rates vary between 60 and 90% in the 12 studies they included into their analysis (14). Our results, showing complete/near-complete occlusion rates of 68% after 1 year and 83% after 2 years, can be found in that range. In line with previous studies, a vessel branch incorporated in the treated aneurysm was found to be predictive of occlusion failure at 6-month follow-up (17). However, a Kaplan–Meier analysis revealed that a vessel branch covered by the flow diverter only delays aneurysm occlusion. At the 2-year follow-up, aneurysm occlusion rates in aneurysms with vs. without incorporated vessels leveled out. The rate of occlusion or narrowing of a vessel branch covered by FRED Jr was 11% at the 6-month follow-up, 19% at the 1-year follow-up, and 23% at the 2-year follow-up. However, none of the patients in this cohort was symptomatic. A possible explanation is that the diminished flow in an aneurysmal vessel branch after flow diverter placement induces the development of a pial collateralization, slowly reducing the demand for antegrade blood supply, which then facilitates the occlusion of the aneurysm (18, 19). Our study results indicate that occlusion of aneurysms with incorporated vessel branches might take more time but can occur even years after the treatment.

Three of the six aneurysms, which needed retreatment, had a clearly dissecting morphology (20). In dissected vessels, the chronically diseased vessel wall is characterized by a disruption of the endothelium, which might result in an inadequate endothelial re-layering (21). Although flow diverter stent treatment might act as a reconstructive technique for dissected vessels by maintaining parent vessel patency and inducing vessel wall healing, further research will be needed to identify which cases are suitable for this technique. Hence, we should be alert in cases of suspected dissecting aneurysms treated with flow diverter stents, controlling for aneurysm recurrence or growth in shorter intervals (21).

### Limitations and Strengths

Patients for this study were recruited from multiple international centers, which allowed the inclusion of a large number of cases and a broad spectrum of applications for FRED Jr. The focus of this study was to analyze the applicability of FRED Jr, and therefore, aneurysm inclusion was not restricted to a specific aneurysm location or aneurysm shape or type. Nevertheless, the retrospective nature of the study comes with data inhomogeneity and limits the flexibility of statistical analysis of the aneurysm treatments. Furthermore, our study group was very heterogeneous with regard to shape, size, and location of the aneurysms, but most aneurysms were small (<1 cm maximum diameter) and located at the bifurcation of the middle cerebral artery or the A1/anterior communicating artery (ACom) complex. Fusiform and dissecting aneurysms as well as aneurysms from the posterior circulation are underrepresented, and results from our statistical analyses might not be easily applied to these subgroups.

## Conclusions

In this retrospective multicenter study of 159 unruptured or previously treated but recanalized aneurysms, flow diversion with the FRED Jr proved to be technically successful and clinically safe. Adequate aneurysm occlusion rate was 83% after 2 years. Most importantly, interventionalists should not be discouraged by early follow-up results after treatment of aneurysms with an incorporated vessel branch, since they often occlude later in time.

## Data Availability Statement

The original contributions presented in the study are included in the article/Supplementary Material, further inquiries can be directed to the corresponding author/s.

## Ethics Statement

The studies involving human participants were reviewed and approved by Ethikkommission der Medizinischen Fakultät Heidelberg and Etik Kurulu of Cerrahpasa Tip Fakültesi Istanbul. Written informed consent to participate in this study was provided by the participants.

## Author Contributions

NK and MM designed the study. NK, CI, MM, FB, IS, MK-O, and SC performed the interventions, were responsible for patient monitoring, and data acquisition of the post-interventional period. JJ, NA, and EÖ performed image analysis. EÖ, DV, and NA performed review of the clinical information. JJ wrote the paper. OK, NK, MB, DV, EÖ, and IS reviewed the paper. All authors contributed to the article and approved the submitted version.

## Conflict of Interest

NK: Consulting & Proctoring Agreement with Microvention. IS: Consulting & Proctoring Agreement with Medtronic and Microvention. SC: Consulting & Proctoring Agreement with Medtronic and Microvention; shareholder: NDI Technologies, Vesalio Inc., Elum Technologies. CI: Proctoring & Training Agreement with Microvention, NDI cofounder and shareholder, Neuravention INC shareholder. MM has received grants from Balt, Medtronic, MicroVention, and Stryker outside the submitted work. MB reports personal fees from Boehringer Ingelheim, BBraun, Vascular Dynamics, Bayer, Merck, Teva, Grifols, grants and personal fees from Novartis and Guerbet, grants from Siemens, Hopp Foundation from DFG, European Union, Stryker, outside the submitted work. The remaining authors declare that the research was conducted in the absence of any commercial or financial relationships that could be construed as a potential conflict of interest.

## Publisher's Note

All claims expressed in this article are solely those of the authors and do not necessarily represent those of their affiliated organizations, or those of the publisher, the editors and the reviewers. Any product that may be evaluated in this article, or claim that may be made by its manufacturer, is not guaranteed or endorsed by the publisher.

## Abbreviations

ACA: anterior cerebral artery
ACom: anterior communicating artery
ASA: acetylsalicylic acid
CSC: Cekirge-Saatci Classification
FRED Jr: FRED Junior
FDCTA: flat detector computed tomography angiography
NICE: non-ischemic cerebral enhancing
OKM: O'Kelly-Marotta Grading Scale
PCA: posterior cerebral artery
PCom: posterior communicating artery
SUCA: superior cerebellar artery.
