# Supplementary material for: Safety and Efficacy of the FRED Jr Flow Re-Direction Endoluminal Device for Intracranial Aneurysms: Retrospective Multicenter Experience With Emphasis on Midterm Results
Source: Front Neurol. 2021 Oct 1;12:722183. doi: 10.3389/fneur.2021.722183 (PMC8518710; doi:10.3389/fneur.2021.722183)
Supplement: Supplementary file 1 [file Data_Sheet_1.docx]

**Supplementary Material**

Participating centers with Principal Investigators in order of enrollment:

Department of Neuroradiology, Cerrahpasa Medical Faculty, Istanbul University, Istanbul, Turkey. Principal Investigator: Naci Koçer; 58 cases. Two operators.

Department of Neuroradiology, Heidelberg University Hospital, Heidelberg, Germany. Principal Investigator: Markus A. Möhlenbruch; 16 cases. One operator.

Department of Radiology, Marmara University School of Medicine, Istanbul, Turkey. Principal Investigator: Feyyaz Baltacioglu; 44 cases. One operator.

Department of Radiology, Yüksek Ihtisas University, Koru Hospital, Ankara, Turkey. Principal Investigator: Isil Saatci; 16 cases. One operator.

Department of Neurology/ Research Institute of Neurointervention, Paracelsus Medical University, Salzburg, Austria. Principal Investigator: Monika Killer-Oberpfalzer; 9 cases. One operator.

Department of Radiology, Koru and Bayindir Hospital, Ankara, Turkey. Principal Investigator: Saruhan Cekirge; 16 cases. One operator.

**Online Table 1: Patient Demographics and Aneurysm Features**

| **A No.** | **P No.** | **Location** | **Type** | **Size (mm)** | **Neck (mm)** | **PV Size p/d, (mm)** | **FRED Jr Size^a^** | **Coil/ PTA** | **Anti-platelets** |
| --- | --- | --- | --- | --- | --- | --- | --- | --- | --- |
| 1 | 1 | MCA bifur | F | 7 | NA | 2.6/1.5 | 2526 | N/N | ASA/clopi. |
| 2 | 2 | ICA tip | S | 6 | 7 | 2.9/2.9 | 3021 | N/N | prasugrel |
| 3 | 3 | MCA M1 | D | 6 | NA | 1.9/2.2 | 3014 | N/Y | ASA/clopi. |
| 4 | 4 | ACA A1/A2 | S | 3 | 2.4 | 2.6/2.6 | 3009 | N/N | ASA/clopi. |
| 5 | 5 | ACA A2/A3 | S | 8 | 3 | 1.4/1.6 | 2520 | N/N | prasugrel |
| 6 | 6 | MCA bifur | S | 5 | 2.6 | 2.8/2.1 | 2520 | N/N | prasugrel |
| 7 | 7 | MCA M1 | S | 8.8 | 4.1 | 2.6/2.0 | 3021 | N/N | ASA/clopi. |
| 8 | 8 | AcomA | S | 8 | 5 | 2.4/1.8 | 2526 | N/N | prasugrel |
| 9 | 9 | ACA A2/A3 | F | 10.5 | NA | 2.6/2.3 | 2513 | N/N | prasugrel |
| 10 | 10 | AcomA | S | 11 | 5 | 2.2/2.2 | 3027 | N/N | prasugrel |
| 11 | 11 | AcomA | S | 12 | 7 | 2.1/2.6 | 2526 | Y/N | prasugrel |
| 12 | 12 | MCA M2 | S | 4 | 1.5 | 2.1/2.3 | 2520 | N/N | ASA/clopi. |
| 13 | 13 | ACA A2/A3 | S | 5 | 3.5 | 3.0/3.4 | 3021 | N/N | ASA/clopi. |
| 14 | 14 | MCA bifur | F | 7.4 | 5.8 | 2.1/1.7 | 3014 | N/N | ASA/clopi. |
| 15 | 15 | ACA A2/A3 | F | 12.2 | NA | 2.2/2.0 | 2520 | N/N | ASA/clopi. |
| 16 | 16 | MCA M1 | S | 3.5 | 3 | 2.5/2.2 | 2508 | N/N | ASA/clopi. |
| 17 | 17 | MCA M2 | S | 3.9 | 2.3 | 2.9/3.0 | 3014 | N/N | ASA/clopi. |
| 18 | 18 | MCA M1 | S | 4.2 | 3.1 | 2.8/2.4 | 3014 | N/N | ASA/clopi. |
| 19 | 19a | ACA A2/A3 | S | 5 | 4.4 | 2.0/2.2 | 2520 | N/N | prasugrel |
| 20 | 19b | ACA A1/A2 | S | 2.3 | 1.7 | 2.3/2.3 | 2513 | N/N | prasugrel |
| 21 | 20 | ACA A2/A3 | S | 2.3 | 2.4 | 2.1/1.8 | 2520 | N/N | prasugrel |
| 22 | 21 | SCA | S | 6.2 | 4 | 3.4/2.6 | 3009 | N/N | prasugrel |
| 23 | 22 | ACA A2/A3 | B | 1.3 | 1.3 | 2.7/2.3 | 3014 | N/N | prasugrel |
| 24 | 23 | ACA A2/A3 | S | 6.8 | 5.9 | 2.2/1.7 | 2526 | N/N | prasugrel |
| 25 | 24 | MCA bifur | S | 11 | 8 | 2.0/1.8 | 2520 | N/N | prasugrel |
| 26 | 25 | ACA A3 | S | 4 | 3 | 2.5/1.9 | 2520 | N/N | prasugrel |
| 27 | 26 | MCA bifur | S | 4.5 | 3.4 | 3.0/2.3 | 3021 | N/N | prasugrel |
| 28 | 27 | ACA A2/A3 | S | 7.5 | 4.2 | 2.5/1.9 | 3014 | N/N | ASA/clopi. |
| 29 | 28 | MCA bifur | G | 25 | 11 | 3.2/1.9 | 3021 | N/N | ASA/clopi. |
| 30 | 29 | ACA A1/A2 | S | 4 | 3 | 1.7/2.2 | 3014 | N/N | ASA/clopi. |
| 31 | 30 | MCA bifur | S | 6.5 | 6 | 2.3/2.2 | 3021 | N/N | ASA/clopi. |
| 32 | 31 | MCA bifur | F | 6.9 | 4.7 | 2.4/2.7 | 2520 | N/N | ASA/clopi. |
| 33 | 32 | ACA A2/A3 | S | 5.8 | 4.6 | 2.4/1.7 | 2520 | N/N | ASA/clopi. |
| 34 | 33 | PCA P1/P2 | S | 11 | 4.8 | 1.8/1.7 | 2513 | N/N | ASA/clopi. |
| 35 | 34 | AcomA | S | 5 | 4.3 | 2.0/2.0 | 2520 | N/N | ASA/clopi. |
| 36 | 35 | PCA P2/P3 | G | 16.1 | 5.5 | 1.7/2.1 | 2520 | N/N | ASA/clopi. |
| 37 | 36 | ACA A2/A3 | S | 3.9 | 2.2 | 2.1/1.8 | 2508 | N/N | ASA/clopi. |
| 38 | 37 | ACA A1/A2 | S | 7.2 | 4.6 | 2.9/2.8 | 3014 | N/N | ASA/clopi. |
| 39 | 38 | ACA A2/A3 | S | 7.8 | 4.9 | 1.7/1.6 | 2513 | N/N | ASA/clopi. |
| 40 | 39 | VA V4 | D | 17.2 | 14.5 | 3.6/1.8 | 3027 | N/N | ASA/clopi. |
| 41 | 40 | PCA P2 | F | 14 | NA | 2.0/1.6 | 3027 | N/N | ASA/clopi. |
| 42 | 41 | MCA M3 | S | 6.2 | 4.5 | 1.7/1.5 | 2513 | N/N | ASA/clopi. |
| 43 | 42 | PCA P2 | D | 36 | NA | 2.9/2.3 | 3027 | N/N | prasugrel |
| 44 | 43 | MCA bifur | S | 5 | 3 | 2.3/1.8 | 2513 | N/N | prasugrel |
| 45 | 44 | ACA A2/A3 | S | 5 | 2.5 | 2.1/2.1 | 2513 | N/N | prasugrel |
| 46 | 45 | MCA bifur | S | 13 | 6 | 2.7/2.4 | 3021 | N/N | prasugrel |
| 47 | 46 | MCA M3 | S | 14 | 5.8 | 2.2/1.6 | 3021 | N/N | prasugrel |
| 48 | 47 | ACA A2/A3 | S | 6 | 5.5 | 2.2/1.8 | 2520 | N/N | prasugrel |
| 49 | 48 | AcomA | S | 3 | 3 | 2.1/1.8 | 2513 | N/N | ASA/clopi. |
| 50 | 49 | MCA M1 | F | 9 | 3 | 2.6/1.7 | 3522 | N/N | ASA/clopi. |
| 51 | 50 | AcomA | S | 5 | 4 | 1.8/1.7 | 3019 | Y/N | ASA/clopi. |
| 52 | 51 | MCA bifur | S | 8.6 | 5.1 | 2.5/2.2 | 3514 | N/N | prasugrel |
| 53 | 52 | ACA A1/A2 | S | 3.7 | 3.1 | 3.0/2.1 | 3014 | N/N | prasugrel |
| 54 | 53 | MCA bifur | S | 9.9 | 3.2 | 2.2/2.2 | 3021 | N/N | prasugrel |
| 55 | 54 | MCA bifur | S | 6 | 5 | 2.6/2.1 | 3014 | N/N | prasugrel |
| 56 | 55 | AcomA | G | 9 | 6 | 3.3/2.0 | 3027 | N/N | ASA/clopi. |
| 57 | 56 | ACA A2/A3 | S | 5.3 | 4.5 | 2.1/1.9 | 2520 | N/N | ASA/clopi. |
| 58 | 57 | ACA A1/A2 | F | 12 | 10 | 2.5/2.0 | 3027 | N/N | prasugrel |
| 59 | 58 | MCA bifur | S | 6.7 | 5 | 3.2/2.5 | 3014 | N/N | prasugrel |
| 60 | 59 | MCA bifur | D | 9 | 6.5 | 2.7/1.9 | 3014 | N/N | prasugrel |
| 61 | 60 | MCA bifur | F | 5 | 2.8 | 2.8/1.8 | 3014 | N/N | prasugrel |
| 62 | 61 | ACA A2/A3 | S | 3.1 | 2.3 | 2.5/2.0 | 2520 | N/N | prasugrel |
| 63 | 62 | MCA bifur | S | 4.2 | 2.5 | 2.0/2.0 | 2513 | N/N | prasugrel |
| 64 | 63a | MCA bifur | S | 7 | 6 | 2.3/2.3 | 3014 | N/N | ASA/clopi. |
| 65 | 63b | MCA bifur | S | 5.5 | 3.8 | 2.7/2.4 | 3014 | N/N | ASA/clopi. |
| 66 | 64 | AcomA | S | 2.3 | 1.6 | 2.2/2.1 | 3014 | N/N | prasugrel |
| 67 | 65 | MCA bifur | S | 6 | 3.5 | 2.3/1.5 | 3014 | N/N | prasugrel |
| 68 | 66 | ACA A1/A2 | S | 5 | 3.5 | 2.8/2.1 | 3014 | N/N | prasugrel |
| 69 | 67 | AcomA | S | 2.7 | 1.1 | 2.0/1.9 | 3014 | N/N | ASA/clopi. |
| 70 | 68 | MCA bifur | S | 6.7 | 3.8 | 2.4/2.1 | 3021 | N/N | ASA/clopi. |
| 71 | 69 | MCA M3 | D | 5.5 | 6 | 1.9/1.7 | 3021 | N/N | prasugrel |
| 72 | 70 | VA V4 | D | 16 | 8 | 3.2/2.6 | 3027 | Y/N | ASA/clopi. |
| 73 | 71 | VA V4 | F | 7 | 7 | 2.4/1.8 | 3021 | N/N | ASA/clopi. |
| 74 | 72 | ACA A2/A3 | S | 2.7 | 1.7 | 2.1/2.1 | 3013 | N/N | ASA/clopi. |
| 75 | 73 | ACA A2/A3 | S | 5.1 | 2.3 | 2.2/1.9 | 2513 | N/N | ASA/clopi. |
| 76 | 74 | PCA P1 | S | 3.2 | 1.8 | 1.8/1.8 | 2508 | N/N | ASA/clopi. |
| 77 | 75a | ACA A1/A2 | S | 6.5 | 4 | 1.9/1.9 | 2508 | N/N | ASA/clopi. |
| 78 | 75b | MCA bifur | S | 3.1 | 2.8 | 2.1/1.8 | 2508 | N/N | ASA/clopi. |
| 79 | 76 | PCA P3 | D | 15 | NA | 2.2/2.0 | 2526 | N/N | ASA/clopi. |
| 80 | 77 | MCA M1 | D | 8.8 | 5.7 | 3.3/2.7 | 3011 | N/N | ASA/clopi. |
| 81 | 78 | MCA bifur | S | 4.1 | 3 | 2.8/2.2 | 3009 | N/N | ASA/clopi. |
| 82 | 79 | PCA P3 | D | 5.7 | 5.6 | 1.7/1.6 | 2513 | N/N | prasugrel |
| 83 | 80 | MCA M2 | S | 6.2 | 4.6 | 2.3/1.9 | 2513 | N/N | ASA/clopi. |
| 84 | 81 | MCA bifur | D | 2.4 | 2.4 | 2.5/2.4 | 2513 | N/N | prasugrel |
| 85 | 82 | MCA M1 | S | 3.5 | 3.2 | 2.7/2.5 | 3014 | N/N | prasugrel |
| 86 | 83a | ACA A1/A2 | S | 3 | 2.9 | 1.7/1.9 | 3014 | N/N | ASA/clopi. |
| 87 | 83b | MCA bifur | S | 6.7 | 6 | 2.2/1.6 | 3014 | N/N | ASA/clopi. |
| 88 | 84 | PCA SUCA | S | 3.1 | 6.5 | 2.2/1.9 | 3014 | N/N | prasugrel |
| 89 | 85 | ACA A2/A3 | S | 7.8 | 6.5 | 2.2/2.3 | 3021 | N/N | prasugrel |
| 90 | 86 | MCA bifur | S | 4 | 2.8 | 2.6/2.4 | 3009 | N/N | prasugrel |
| 91 | 87 | MCA bifur | S | 4.6 | 4.8 | 2.5/2.5 | 3014 | N/N | ASA/clopi. |
| 92 | 88 | MCA M1 | S | 8.8 | 7.1 | 2.8/2.5 | 3014 | N/N | prasugrel |
| 93 | 89 | MCA M1 | S | 4.5 | 1.7 | 1.8/1.6 | 2513 | N/N | ASA/clopi. |
| 94 | 90 | AcomA | S | 4.6 | 3.1 | 2.5/1.9 | 3014 | N/N | ASA/clopi. |
| 95 | 91 | MCA bifur | S | 4 | 2.4 | 2.4/2.0 | 3009 | N/N | prasugrel |
| 96 | 92a | MCA M1 | S | 4.6 | 2.9 | 2.4/2.2 | 2513 | N/N | ASA/clopi. |
| 97 | 92b | ACA A1/A2 | S | 2.9 | 1.7 | 2.1/1.8 | 2513 | N/N | ASA/clopi. |
| 98 | 93 | AcomA | S | 4 | 2.6 | 2.3/1.7 | 2520 | N/N | ASA/clopi. |
| 99 | 94 | PCA P3 | D | 15 | 8.3 | 2.0/1.7 | 2520 | Y/N | prasugrel |
| 100 | 95 | MCA bifur | S | 4.7 | 3.5 | 2.9/2.6 | 3009 | N/N | prasugrel |
| 101 | 96 | MCA M2 | S | 4.3 | 3.2 | 2.9/2.9 | 3009 | N/N | prasugrel |
| 102 | 97a | MCA M1 | S | 3 | 2.2 | 2.4/2.1 | 3014 | N/N | prasugrel |
| 103 | 97b | ACA A1/A2 | S | 1.5 | 1.5 | 2.2/2.0 | 2513 | N/N | prasugrel |
| 104 | 98 | PCA P3 | S | 3.8 | 2.2 | 2.2/1.5 | 2513 | N/N | prasugrel |
| 105 | 99 | MCA M1 | S | 5.9 | 3.9 | 2.7/2.5 | 3014 | N/N | ASA/clopi. |
| 106 | 100a | ACA A2 | S | 4.4 | 3.6 | 2.1/1.8 | 2513 | N/N | ASA/clopi. |
| 107 | 100b | ACA A2 | S | 6.5 | 2.5 | 2.0/1.9 | 2520 | N/N | ASA/clopi. |
| 108 | 101 | PCA P2 | S | 11 | 9.1 | 2.5/2.3 | 3021 | Y/N | prasugrel |
| 109 | 102 | MCA M1 | S | 3.2 | 2 | 2.7/3.2 | 3009 | N/N | prasugrel |
| 110 | 103 | ACA A2 | S | 4 | 2.1 | 1.9/2.0 | 2520 | N/N | ASA/clopi. |
| 111 | 104 | MCA bifur | S | 4.6 | 3.8 | 2.7/2.4 | 3014 | N/N | prasugrel |
| 112 | 105 | ACA A2 | S | 6.8 | 4 | 1.9/2.1 | 2520 | N/N | prasugrel |
| 113 | 106 | MCA M1 | S | 9 | 6.8 | 2.5/2.3 | 3014 | N/N | prasugrel |
| 114 | 107 | ACA A1/A2 | S | 6.2 | 3.5 | 2.2/2.3 | 3014 | N/N | prasugrel |
| 115 | 108 | MCA bifur | S | 7.7 | 3.7 | 2.8/2.5 | 3014 | N/N | ASA/clopi. |
| 116 | 109 | MCA M1 | S | 5.3 | 1.7 | 2.6/2.5 | 3009 | N/N | prasugrel |
| 117 | 110a | ACA A2/A3 | S | 4.4 | 2.7 | 2.4/2.4 | 2513 | N/N | prasugrel |
| 118 | 110b | MCA bifur | S | 8.2 | 3.1 | 2.9/2.1 | 3014 | N/N | prasugrel |
| 119 | 111 | AcomA | S | 3.3 | 2.7 | 2.1/2.0 | 3014 | N/N | ASA/clopi. |
| 120 | 112 | MCA M1 | S | 4.7 | 3.5 | 2.3/2.1 | 3009 | N/N | ASA/clopi. |
| 121 | 113 | MCA bifur | S | 2.9 | 1.8 | 2.5/2.2 | 3014 | N/N | ASA/clopi. |
| 122 | 114 | ACA A2/A3 | S | 3 | 1.8 | 1.7/1.6 | 2508 | N/N | prasugrel |
| 123 | 115 | MCA M3 | D | 19 | NA | 2.0/1.4 | 2526 | N/N | prasugrel |
| 124 | 116 | AcomA | S | 11 | 4.4 | 2.5/2.1 | 2508 | N/N | prasugrel |
| 125 | 117 | MCA bifur | S | 4.5 | 4 | 2.4/1.6 | 3014 | N/N | prasugrel |
| 126 | 118 | MCA M1 | D | 1.5 | 1.5 | 2.6/2.0 | 3014 | N/N | prasugrel |
| 127 | 119 | MCA bifur | S | 15.5 | 6.7 | 2.8/2.3 | 3027 | N/N | prasugrel |
| 128 | 120 | ACA A2 | D | 4 | 3 | 2.8/2.2 | 3019 | N/N | prasugrel |
| 129 | 121 | ACA A2 | S | 12 | 8 | 2.8/2.2 | 3027 | N/N | prasugrel |
| 130 | 122 | MCA bifur | S | 3 | 2 | 2.4/2.1 | 2518 | N/N | prasugrel |
| 131 | 123 | AcomA | S | 2 | 2 | 3.0/2.4 | 3027 | N/N | prasugrel |
| 132 | 124 | MCA bifur | S | 4 | 3 | 3.0/2.4 | 3019 | N/N | prasugrel |
| 133 | 125 | MCA bifur | S | 3 | 2 | 2.5/2.1 | 2518 | N/N | prasugrel |
| 134 | 126 | MCA bifur | S | 10 | 6 | 3.0/2.6 | 3019 | N/N | prasugrel |
| 135 | 127 | ACA A2/A3 | S | 4 | 2.5 | 2.5/2.3 | 2518 | N/N | prasugrel |
| 136 | 128 | ACA A1 | B | NA | NA | 2.9/2.6 | 3019 | N/N | prasugrel |
| 137 | 129 | MCA bifur | S | 13 | 4 | 2.8/2.4 | 3013 | N/N | prasugrel |
| 138 | 130 | MCA bifur | S | 5 | 3 | 2.4/2.1 | 2518 | N/N | prasugrel |
| 139 | 131 | MCA bifur | S | 12 | 4 | 2.7/1.8 | 3013 | N/N | prasugrel |
| 140 | 132 | MCA bifur | G | 26 | 6.5 | 2.3/1.9 | 2513 | Y/N | prasugrel |
| 141 | 133 | ACA A1 | D | 5 | 2 | 2.4/2.1 | 2518 | N/N | prasugrel |
| 142 | 134 | MCA bifur | F | 0 | NA | 2.4/2.0 | 2518 | N/N | prasugrel |
| 143 | 135a | AcomA | S | 3 | 3 | 2.9/2.3 | 3019 | N/N | prasugrel |
| 144 | 135b | ACA A2/A3 | S | 2 | 2 | 2.3/2.1 | 2518 | N/N | prasugrel |
| 145 | 136 | PCA P1 | B | 0 | NA | 2.1/2.1 | 2513 | N/N | prasugrel |
| 146 | 137 | ACA A2/A3 | S | 7 | 2 | 2.4/2.1 | 2513 | N/N | prasugrel |
| 147 | 138 | MCA bifur | S | 13 | 5 | 2.4/2.2 | 2525 | N/N | prasugrel |
| 148 | 139 | AcomA | S | 19 | 6 | 2.8/2.3 | 3027 | N/N | ASA/clopi. |
| 149 | 140 | MCA bifur | S | 5 | 4 | 2.4/2.1 | 2513 | N/N | prasugrel |
| 150 | 141 | MCA M3 | S | 3 | 2.5 | 2.3/2.0 | 2513 | N/N | prasugrel |
| 151 | 142 | MCA bifur | S | 3 | 2 | 2.8/2.4 | 3019 | N/N | prasugrel |
| 152 | 143 | AcomA | S | 4.6 | 4.9 | 2.1/2.1 | 2513 | N/N | prasugrel |
| 153 | 144 | PcomA | S | 4 | 4 | 2.1/1.8 | 3027 | N/N | ASA/clopi. |
| 154 | 145 | MCA bifur | S | 3 | 3 | 2.2/1.6 | 2513 | N/N | ASA/clopi. |
| 155 | 146 | Acom | S | 3 | 3 | 2.4/1.7 | 2525 | Y/N | ASA/clopi. |
| 156 | 147 | ACA A2 | S | 6 | 3 | 2.0/1.8 | 2513 | N/N | ASA/clopi. |
| 157 | 148 | ACA A1 | S | 4 | 2.9 | 2.1/1.8 | 2513 | N/N | prasugrel |
| 158 | 149 | AcomA | S | 3 | 3 | 1.8/1.5 | 2525 | N/N | ASA/clopi. |
| 159 | 150 | MCA M3 | F | 7 | 4 | 2.1/1.9 | 2513 | N/N | ASA/clopi. |

**Note**: A, aneurysm; AcomA, anterior communicating artery; B, blisterlike; clopi., clopidogrel; D, dissecting; F, fusiform; G, giant; bifur, bifurcation; NA, not applicable; No., number; P, patient; PCA, posterior cerebral artery; PcomA, posterior communicating artery; p/d, proximal/ distal; PTA, percutaneous transluminal angioplasty; PV, parent vessel; S, saccular; SCA, superior cerebellar artery; VA, vertebral artery; Y, yes; N, no. ^a^ The first two digits refer to the device diameter, and the last two digits represent the device working length.

**Online Table 2: Clinical, Angiographic and Follow-up Details**

| **A No.** | **P No.** | **Clinical Presentation** | **Additional information**  **(treatment and first 30 days)** | **Additional information (FU)** |
| --- | --- | --- | --- | --- |
| 1 | 1 | incidental, regrowth after previous clipping | - | **-** |
| 2 | 2 | incidental, regrowth after previous coiling | - | - |
| 3 | 3 | incidental, headaches | Technical: Insufficient opening of distal part of the FRED Jr, therefore PTA was performed.  Minor stroke: 2hr post procedure, transient hemiparesis due to lower trunk MCA occlusion; after i.v. tirofiban, lower trunk reopened, and the symptoms regressed | - |
| 4 | 4 | incidental, headaches | - | - |
| 5 | 5 | initially SAH, remnant after coiling | - | - |
| 6 | 6 | incidental, headaches | - | - |
| 7 | 7 | incidental, headaches | Minor stroke: Stupor for several hours after extubation; MRI findings normal | - |
| 8 | 8 | incidental, headaches | - | - |
| 9 | 9 | incidental | - | - |
| 10 | 10 | incidental | - | - |
| 11 | 11 | incidental, headaches | - | - |
| 12 | 12 | incidental, headaches, multiple aneurysms | - | - |
| 13 | 13 | incidental, headaches | - | - |
| 14 | 14 | incidental, headaches | - | - |
| 15 | 15 | incidental, headaches | - | - |
| 16 | 16 | incidental, headaches, multiple aneurysms | - | - |
| 17 | 17 | incidental, headaches | - | - |
| 18 | 18 | incidental, headaches | - | - |
| 19 | 19a | incidental, multiple aneurysms | - | - |
| 20 | 19b | incidental | - | Stent deformation at 5 months FU causing <50% stenosis of distal parent vessel, asymptomatic |
| 21 | 20 | initially SAH from another aneurysm, multiple aneurysms | - | Side branch compromised: At 35 months FU callosomarginal artery side branch covered by stent is reduced in caliber, asymptomatic |
| 22 | 21 | incidental, remnant after previous Pipeline | - | - |
| 23 | 22 | initially SAH, remnant after previous Silk | - | - |
| 24 | 23 | initially SAH from another aneurysm, multiple aneurysms | - | - |
| 25 | 24 | Incidental, regrowth after previous coiling | - | - |
| 26 | 25 | incidental, headaches | - | - |
| 27 | 26 | incidental, headaches, multiple aneurysms | - | - |
| 28 | 27 | incidental | - | - |
| 29 | 28 | incidental, regrowth after previous clipping | Thrombus formation: upper trunk MCA occlusion occurred immediately after stent deployment; after i.v. tirofiban, upper trunk reopened | - |
| 30 | 29 | initially SAH, regrowth after clipping, multiple aneurysms | - | - |
| 31 | 30 | initially SAH, regrowth after coiling, multiple aneurysms | - | - |
| 32 | 31 | incidental, headaches, multiple aneurysms | - | NICE lesions: at 35 months FU DWI lesions in MCA territory, asymptomatic |
| 33 | 32 | incidental, headaches | - | - |
| 34 | 33 | incidental, discovered during the workup of unrelated stroke | - | - |
| 35 | 34 | incidental, reperfusion after previous coiling | Technical: first stent chosen was 2.5/1.3, decided to be too short, took out before deployment and placed a longer one | - |
| 36 | 35 | symptomatic with transient arm paresis | - | - |
| 37 | 36 | incidental, multiple aneurysms | Technical: First FRED Jr 2.5/1.3 dropped proximal to the aneurysm after removal of the catheter and was snared out with no further complication. Then longer FRED Jr was deployed.  Thrombus formation: immediately after deployment diminished flow in the adjacent branches with complete regression after i.v. tirofiban | NICE lesions: at 4 months FU multiple enhancing DWI lesions in brain parenchyma with regression after corticoid therapy, asymptomatic |
| 38 | 37 | incidental | Minor stroke: transient hemiparesis after extubation due to embolic DWI lesions distal to the FRED Jr with complete regression at discharge | - |
| 39 | 38 | incidental, headaches | - | - |
| 40 | 39 | probably related stroke due to vessel dissection 5 months before treatment, then growing aneurysm in distal vertebral artery | - | 2^nd^ treatment: 24 months after initial FRED Jr treatment a FRED was telescopically placed into the first stent due to insufficient aneurysm occlusion |
| 41 | 40 | incidental, headaches | - | - |
| 42 | 41 | incidental, headaches | - | 2^nd^ treatment: At 1-month FU migration and shortening of FRED Jr into the aneurysm was observed, which led to incomplete coverage of the aneurysm, therefore another FRED JR stent (2525) was telescopically placed into the first stent |
| 43 | 42 | incidental | - | 2^nd^ treatment: At 3 months FU further aneurysm growth, therefore parent artery occlusion by coiling, patient worsened after 2^nd^ treatment with mRS 2 |
| 44 | 43 | incidental | - | - |
| 45 | 44 | incidental, headaches, multiple aneurysms | - | - |
| 46 | 45 | incidental, headaches | - | - |
| 47 | 46 | incidental, headaches | - | - |
| 48 | 47 | incidental, headaches | - | - |
| 49 | 48 | initially SAH, regrowth after coiling | - | - |
| 50 | 49 | incidental | Major stroke: after extubation patient had hemiparesis (mRS 2); the selected FRED Jr seemed too long and compromised the perfusion in one M2 branch; MRI control showed watershed infarctions | - |
| 51 | 50 | initially SAH, regrowth after coiling | - | - |
| 52 | 51 | incidental, vertigo | - | - |
| 53 | 52 | incidental, perioral paresthesia | - | - |
| 54 | 53 | incidental, headaches | - | - |
| 55 | 54 | initially SAH, regrowth after coiling, multiple aneurysms | - | - |
| 56 | 55 | initially SAH, regrowth after coiling | - | - |
| 57 | 56 | incidental, headaches | - | - |
| 58 | 57 | incidental, headaches | Technical: Insufficient opening of first FRED Jr 3027 in the middle portion, second smaller FRED Jr 2527 opened as expected | - |
| 59 | 58 | incidental, headaches | - | - |
| 60 | 59 | symptomatic with right arm paresthesia | - | - |
| 61 | 60 | incidental, vertigo | - | - |
| 62 | 61 | incidental | - | - |
| 63 | 62 | incidental, headaches | - | - |
| 64 | 63a | Initially SAH, remnant after coiling | - | - |
| 65 | 63b | incidental | - | - |
| 66 | 64 | incidental | - | - |
| 67 | 65 | incidental | - | - |
| 68 | 66 | incidental, regrowth after coiling, multiple aneurysms | Pseudoaneurysm in the groin needed surgical treatment | - |
| 69 | 67 | incidental | - | - |
| 70 | 68 | initially SAH, regrowth after coiling, multiple aneurysms | - | - |
| 71 | 69 | incidental, headaches | - | - |
| 72 | 70 | symptomatic, visual and gait disturbance | - | - |
| 73 | 71 | symptomatic, visual disturbance and vertigo | Thrombus formation: temporary flow reduction of the PICA, tirofiban i.v., patient asymptomatic and no long-term sequelae | - |
| 74 | 72 | previous SAH from another aneurysm, multiple aneurysms | - | - |
| 75 | 73 | previous SAH from another aneurysm, multiple aneurysms | - | - |
| 76 | 74 | incidental, multiple aneurysms | - | - |
| 77 | 75a | incidental, discovered during the workup of suspected MS, multiple aneurysms | Thrombus formation: temporary thrombosis of contralateral A2, right A1 - aplasia, hemodynamic microemboli, tirofiban i.v. during procedure and continued for 24 hours, instant regression of thrombi, asymptomatic | - |
| 78 | 75b | incidental | Minor stroke: small ACM infarction 10d after treatment, transient mild paresis of left hand;  Stent deformation: in DSA 10 days after treatment minimal stent deformation with distal caliber reduction of FRED Jr | - |
| 79 | 76 | symptomatic, severe vertigo and headache | Thrombus formation: temporary occlusion of FRED Jr after deployment, tirofiban i.v., vessel recanalization after 10 minutes | - |
| 80 | 77 | symptomatic, ischemia in vessel territory distal of the aneurysm, transient paresis of the hand | - | - |
| 81 | 78 | incidental | - | - |
| 82 | 79 | incidental | Technical: first stent chosen was 2508, decided to be too short, took out before deployment and placed a second one | - |
| 83 | 80 | incidental | - | - |
| 84 | 81 | incidental | - | - |
| 85 | 82 | incidental | - | - |
| 86 | 83a | incidental, multiple aneurysms | Thrombus formation: flow reduction in ACA and MCA, tirofiban i.v.,  MRI showed infarction in the right frontal lobe, asymptomatic. | - |
| 87 | 83b | incidental, regrowth after previous Web-embolization | - | - |
| 88 | 84 | incidental, multiple aneurysms | Thrombus formation: in stent wall: tirofiban i.v.  Minor stroke: Patient was disoriented but improved without sequelae. MRI showed embolic infarctions. | - |
| 89 | 85 | incidental | - | - |
| 90 | 86 | incidental, regrowth after previous clipping | - | - |
| 91 | 87 | incidental | - | - |
| 92 | 88 | incidental, regrowth after previous stent-assisted (Acandis) coiling | - | - |
| 93 | 89 | incidental | Thrombus formation: occlusion of frontal vessel branch, and partial thrombosis in the stent, tirofiban i.v. then instant regression of thrombosis.  Minor stroke: facial paralysis, loss of strength on the right arm and leg after treatment. MRI showed embolic frontal infarction. | - |
| 94 | 90 | incidental | - | - |
| 95 | 91 | incidental, multiple aneurysms | - | - |
| 96 | 92a | incidental, multiple aneurysms | - | - |
| 97 | 92b | incidental | - | - |
| 98 | 93 | previous SAH from another aneurysm, multiple aneurysms | - | - |
| 99 | 94 | incidental, multiple aneurysms | - | 2^nd^ treatment: 29 months after first treatment further aneurysm sac growth, therefore parent artery occlusion by coiling |
| 100 | 95 | incidental | - | - |
| 101 | 96 | incidental | - | - |
| 102 | 97a | incidental, multiple aneurysms | Thrombus formation: Simultaneous treatment of MCA and ACA aneurysm. A partial thrombus that did not disrupt the flow was observed in both stents, tirofiban i.v., instant thrombus regression | - |
| 103 | 97b | incidental | - | - |
| 104 | 98 | incidental, multiple aneurysms | - | - |
| 105 | 99 | incidental, multiple aneurysms | - | - |
| 106 | 100a | incidental, multiple aneurysms | - | - |
| 107 | 100b | incidental | - | - |
| 108 | 101 | incidental | - | - |
| 109 | 102 | incidental, multiple aneurysms | - | - |
| 110 | 103 | incidental, multiple aneurysms, Alzheimer disease | - | - |
| 111 | 104 | incidental | - | - |
| 112 | 105 | incidental, multiple aneurysms | - | - |
| 113 | 106 | incidental, multiple aneurysms | - | - |
| 114 | 107 | incidental | - | - |
| 115 | 108 | incidental, multiple aneurysms | Thrombus formation: acute in-stent thrombosis, tirofiban i.v., instant regression | - |
| 116 | 109 | incidental, multiple aneurysms | - | - |
| 117 | 110a | incidental, multiple aneurysms | - | - |
| 118 | 110b | incidental | - | - |
| 119 | 111 | incidental, multiple aneurysms | - | - |
| 120 | 112 | incidental, multiple aneurysms | - | - |
| 121 | 113 | incidental, headaches | - | - |
| 122 | 114 | incidental, headaches, multiple aneurysms | - | - |
| 123 | 115 | initially SAH, regrowth after clipping | Minor SAH during procedure | - |
| 124 | 116 | incidental, headaches | - | - |
| 125 | 117 | incidental, headaches, vertigo | - | - |
| 126 | 118 | incidental, headaches | - | - |
| 127 | 119 | incidental | - | - |
| 128 | 120 | symptomatic with stroke due to dissection of ACA | - | - |
| 129 | 121 | incidental, headaches | - | 2^nd^ treatment: 8 months after initial treatment unchanged aneurysm and patient suffering from severe headache, therefore retreatment with telescopic placement of a 2nd FRED Jr, small and asymptomatic SAH in postop DYNA-CT without further clinical consequence |
| 130 | 122 | previous sah from another aneurysm, multiple aneurysms | - | - |
| 131 | 123 | previous sah from another aneurysm, multiple aneurysms | Technical: First FRED Jr 1419 dropped proximal to the aneurysm after removal of the catheter and was snared out with no further complication, longer one was placed | - |
| 132 | 124 | incidental, regrowth after coiling | - | - |
| 133 | 125 | incidental, headaches | - | - |
| 134 | 126 | initially SAH, regrowth after stent-assisted (ATLAS) coiling | - | - |
| 135 | 127 | incidental, multiple aneurysms | - | 2^nd^ treatment: 25 months after initial treatment unchanged aneurysm, retreatment with telescopic placement of a Pipeline |
| 136 | 128 | incidental | Thrombus formation: acute in-stent thrombosis, tirofiban i.v. + LVIS Jr placed within Fred Jr, then complete recanalization | - |
| 137 | 129 | incidental, regrowth after previous embolization by Web and Pipeline | - | - |
| 138 | 130 | incidental after previous SAH, multiple aneurysms | - | branch originating from aneurysm occluded: At 13 months FU inferior trunk of MCA artery side branch covered by stent is occluded and filling retrogradely, asymptomatic |
| 139 | 131 | incidental, headaches | - | - |
| 140 | 132 | headaches, brain edema adjacent to aneurysm sac, treatment after failed clipping | - | - |
| 141 | 133 | incidental, headaches | - | - |
| 142 | 134 | incidental, headaches | - | - |
| 143 | 135a | incidental, multiple aneurysms | - | - |
| 144 | 135b | incidental | - | - |
| 145 | 136 | incidental, headaches | - | - |
| 146 | 137 | initially SAH, remnant after coiling | - | - |
| 147 | 138 | initially SAH, regrowth after clipping | - | - |
| 148 | 139 | incidental, regrowth after coiling | Minor stroke: 2nd day after treatment, right upper extremity weakness under ticagrelor and ASA, cleared after addition of clexane without sequelae | - |
| 149 | 140 | incidental, headaches | - | - |
| 150 | 141 | incidental after previous SAH, multiple aneurysms | - | - |
| 151 | 142 | incidental, headaches | - | - |
| 152 | 143 | incidental | - | - |
| 153 | 144 | incidental, tinnitus | - | - |
| 154 | 145 | incidental after previous SAH, multiple aneurysms, regrowth after coiling | - | - |
| 155 | 146 | initially SAH, regrowth after coiling | - | - |
| 156 | 147 | incidental | - | - |
| 157 | 148 | incidental, pituitary prolactinoma | Patient showed to be a clopidogrel and ticagrelor non-responder and was switched to prasugrel | branch associated with aneurysm located at a vessel fenestration occluded as intended at 2 months FU |
| 158 | 149 | initially SAH, regrowth after coiling | Thrombus formation: acute in-stent thrombosis, tirofiban i.v.; at the end of procedure both ACA showed flow reduction; patient had no antiplatelet premedication  Major stroke: Infarctions in both ACA territories (mRS 4) | - |
| 159 | 150 | incidental | - | - |

**Note**: A, aneurysm; ACA, anterior cerebral artery; ASA, acetylsalicylic acid; DSA, digital subtraction angiography; DWI, diffusion weighted imaging; FU, follow-up; MCA, middle cerebral artery; mRS, modified Rankin Scale; NICE, non-ischemic contrast-enhancing; P, patient; -, absent; SAH, subarachnoid hemorrhage.

**On-line Table 3: mRS, OKM and CSC at follow-up**

| **A No.** | **P No.** | **mRS baseline 6-/ 12-/ 24-/ 36-months follow-up** | **Aneurysm occlusion (OKM) 6-/ 12-/ 24-/ 36-months follow-up** | **Aneurysm occlusion (CSC) 6-/ 12-/ 24-/ 36- months follow-up** |
| --- | --- | --- | --- | --- |
| 1 | 1 | 0/0/0/0/0 | C/C1/C/C | 2/2/2/2 |
| 2 | 2 | 0/0/0/0/0 | B/B2/C/C | 3/3/3/2 |
| 3 | 3 | 0/0/0/0/0 | D/D/D/D | 1B/1B/1B/1B |
| 4 | 4 | 0/0/0/c.f./c.f. | D/D/c.f./c.f. | 1A/1A/c.f./c.f. |
| 5 | 5 | 0/0/0/0/0 | D/D/D/D | 1A/1A/1A/1A |
| 6 | 6 | 0/0/0/0/0 | D/D/D/D | 1B/1A/1A/1A |
| 7 | 7 | 0/0/0/0/0 | B2/B/B/B | 3/3/3/3 |
| 8 | 8 | 0/0/0/0/0 | B/B/B/B | 3/3/3/3 |
| 9 | 9 | 0/0/0/0/0 | D/D/D/D | 1A/1A/1A/1A |
| 10 | 10 | 0/0/0/0/0 | B/B/B/B | 3/3/3/3 |
| 11 | 11 | 0/0/0/c.f./c.f. | D/D/c.f./c.f. | 1A/1A/c.f./c.f. |
| 12 | 12 | 0/0/0/0/0 | D/D/D/D | 1B/1B/1B/1B |
| 13 | 13 | 0/0/0/c.f./c.f. | B2/C2/c.f./c.f. | 3/2/c.f./c.f. |
| 14 | 14 | 0/0/0/0/0 | C1/C/C/C | 2/2/2/2 |
| 15 | 15 | 0/0/0/0/0 | D/D/D/D | 1/1/1/1 |
| 16 | 16 | 0/0/0/0/0 | B/B/D/D | 3/3/1A/1A |
| 17 | 17 | 0/0/0/0/0 | B2 /C3/C3/C3 | 5/5/5/5 |
| 18 | 18 | 0/0/0/0/0 | B3/C3/D/D | 3/3/1C/1C |
| 19 | 19a | 0/0/0/0/0 | D/D/D/D | 1C/1C/1C/1C |
| 20 | 19b | 0/0/0/0/0 | D/D/D/D | 1/1/1/1 |
| 21 | 20 | 0/0/0/0/0 | B/B/D/D | 3/3/1B/1B |
| 22 | 21 | 0/0/0/0/0 | B/B/C/C | 3/3/2/2 |
| 23 | 22 | 0/0/0/0/0 | D/D/D/D | 1/1/1/1 |
| 24 | 23 | 0/0/0/0/0 | D/D/D/D | 1A/1A/1A/1A |
| 25 | 24 | 0/0/0/0/0 | D/D/D/D | 1/1/1/1 |
| 26 | 25 | 0/0/0/0/0 | D/D/D/D | 1/1/1/1 |
| 27 | 26 | 0/0/0/0/0 | B/B/D/D | 3/3/1A/1A |
| 28 | 27 | 0/0/0/0/0 | A/A/A2/A | 3/3/3/3 |
| 29 | 28 | 0/0/0/0/0 | B/B3/D/D | 3/3/1A/1A |
| 30 | 29 | 0/0/0/0/0 | D/D/D/D | 1C/1C/1C/1C |
| 31 | 30 | 0/0/0/0/0 | A2/A2/C/C | 3/3/2/2 |
| 32 | 31 | 0/0/0/0/0 | B/C/C/C | 3/2/1B/2 |
| 33 | 32 | 0/0/0/0/0 | B/B/B/B | 3/3/3/3 |
| 34 | 33 | 1/1/1/c.f./c.f. | D/D/c.f./c.f. | 1/1/c.f./c.f. |
| 35 | 34 | 0/0/0/0/0 | D/D/D/D | 1/1/1/1 |
| 36 | 35 | 0/0/0/0/0 | D/D/D/D | 1/1/1/1 |
| 37 | 36 | 0/0/0/0/0 | C/C/C/D | 2/2/2/1 |
| 38 | 37 | 0/0/0/0/0 | A/D/D/D | 3/1C/1C/1C |
| 39 | 38 | 0/0/0/0/0 | C/C/C/C | 2/2/2/2 |
| 40 | 39 | 0/0/0/0/0 | B/B/D C/D | 3/3/2/1 |
| 41 | 40 | 0/0/0/0/- | C/C2/C/- | 2/2/2/- |
| 42 | 41 | 0/0/0/0/- | B2/B/B/- | 3/3/3/- |
| 43 | 42 | 0/2/2/-/- | D/D/-/- | 1/1/-/- |
| 44 | 43 | 0/0/0/0/- | B3/B3/C3/- | 3/3/5/- |
| 45 | 44 | 0/0/0/0/- | D/D/D/- | 1A/1A/1A/- |
| 46 | 45 | 0/0/0/-/- | B/D/-/- | 3/1C/-/- |
| 47 | 46 | 0/0/0/0/- | C/C/C/- | 2/2/2/- |
| 48 | 47 | 0/0/0/-/- | B2/D/-/- | 3/1C/-/- |
| 49 | 48 | 0/0/0/0/- | D/D/D/- | 1A/1A/1A/- |
| 50 | 49 | 0/2/2/c.f./c.f. | D1/D/c.f./c.f. | 1A/1A/c.f./c.f. |
| 51 | 50 | 0/0/0/0/0 | D/D/D/D | 1/1/1/1 |
| 52 | 51 | 0/0/0/-/- | B/B/-/- | 3/3/-/- |
| 53 | 52 | 0/0/-/-/- | C/-/-/- | 3/-/-/- |
| 54 | 53 | 0/0/-/-/- | B/-/-/- | 3/-/-/- |
| 55 | 54 | 0/0/-/-/- | B/-/-/- | 3/-/-/- |
| 56 | 55 | 0/0/-/-/- | B/-/-/- | 3/-/-/- |
| 57 | 56 | 0/0/-/-/- | B2/-/-/- | 3/-/-/- |
| 58 | 57 | 0/0/-/-/- | B/-/-/- | 3/-/-/- |
| 59 | 58 | 0/0/-/-/- | B/-/-/- | 3/-/-/- |
| 60 | 59 | 0/0/-/-/- | B/-/-/- | 3/-/-/- |
| 61 | 60 | 0/0/-/-/- | B2/-/-/- | 3/-/-/- |
| 62 | 61 | 0/0/0/0/- | D/D/D/- | 1/1/1/- |
| 63 | 62 | 0/0/-/-/- | C/-/-/- | 2/-/-/- |
| 64 | 63a | 0/0/0/0/- | A/A/A/- | 3/3/3/- |
| 65 | 63b | 0/0/-/-/- | A/-/-/- | 3/-/-/- |
| 66 | 64 | 0/0/0/-/- | D/D/-/- | 1/1/-/- |
| 67 | 65 | 0/0/0/-/- | B/B/-/- | 3/3/-/- |
| 68 | 66 | 0/0/0/-/- | A/D/-/- | 3/1C/-/- |
| 69 | 67 | 0/0/-/-/- | B/-/-/- | 2/-/-/- |
| 70 | 68 | 0/0/0/0/- | B/B1/B/- | 3/3/3/- |
| 71 | 69 | 0/0/0/-/- | D/D/-/- | 1/1/-/- |
| 72 | 70 | 0/0/0/0/- | C/D/D/- | 2/1/1/- |
| 73 | 71 | 0/0/0/0/- | B/C/D/- | 3/2/1/- |
| 74 | 72 | 0/0/0/0/- | B/B/B/- | 3/3/3/- |
| 75 | 73 | 3/3/3/3/- | D/D/D/- | 1A/1A/1A/- |
| 76 | 74 | 1/1/1/-/- | C/C/-/- | 2/2/-/- |
| 77 | 75a | 0/0/0/-/- | D/D/-/- | 1A/1A/-/- |
| 78 | 75b | 0/0/0/-/- | D/D/-/- | 1A/1A/-/- |
| 79 | 76 | 0/0/-/-/- | D/-/-/- | 1A/-/-/- |
| 80 | 77 | 0/0/-/-/- | B/-/-/- | 3/-/-/- |
| 81 | 78 | 0/0/-/-/- | A/-/-/- | 3/-/-/- |
| 82 | 79 | 0/0/0/0/0 | B3/B3/C/C | 3/3/5/5 |
| 83 | 80 | 0/0/0/c.f./c.f. | C3/D/c.f./c.f. | 2/1A/c.f./c.f. |
| 84 | 81 | 0/0/0/0/0 | D/D/D/D | 1/1/1/- |
| 85 | 82 | 0/0/0/0/0 | B2/C2/D/D | 3/5/5/5 |
| 86 | 83a | 0/0/0/0/0 | D/D/D/D | 1/1/1/1 |
| 87 | 83b | 0/0/0/0/0 | B3/B3/B3/B3 | 3/3/3/3 |
| 88 | 84 | 0/0/0/0/0 | B1/B1/C B1/C | 5/5/5/5 |
| 89 | 85 | 0/0/0/0/- | B3/B3/B3/- | 3/3/3/- |
| 90 | 86 | 0/0/0/0/- | B2/B2/B2/- | 3/3/3/- |
| 91 | 87 | 0/0/0/0/- | B2/B2/D/- | 3/5/5/- |
| 92 | 88 | 0/0/0/c.f./- | B3/B3/c.f./- | 3/3/c.f./- |
| 93 | 89 | 0/0/0/c.f./- | B3/B3/c.f./- | 3/3/c.f./- |
| 94 | 90 | 0/0/0/0/- | D/D/D/- | 1/1/1/- |
| 95 | 91 | 0/0/0/0/- | D/D/D/- | 1C/1C/1C/- |
| 96 | 92a | 0/0/0/0/- | A3/B3/B3/- | 3/3/3/- |
| 97 | 92b | 0/0/0/0/- | D/D/D/- | 1/1/1/- |
| 98 | 93 | 0/0/0/0/- | A3/A3/B3/- | 3/3/3/- |
| 99 | 94 | 0/0/0/0/- | B3/B3/C3/- | 3/3/2/- |
| 100 | 95 | 0/0/0/-/- | B3/B3/-/- | 5/5/-/- |
| 101 | 96 | 0/0/0/-/- | D/D/-/- | 1A/1A/-/- |
| 102 | 97a | 0/0/0/0/- | A3/A3/B3/- | 3/3/3/- |
| 103 | 97b | 0/0/0/0/- | D/D/D/- | 1/1/1/- |
| 104 | 98 | 0/0/0/0/- | B2/B3/C/- | 3/5/5/- |
| 105 | 99 | 0/0/0/-/- | C/C/-/- | 5/5/-/- |
| 106 | 100a | 0/0/0/-/- | A3/B3/-/- | 3/3/-/- |
| 107 | 100b | 0/0/0/-/- | C/C/-/- | 5/5/-/- |
| 108 | 101 | 0/0/0/-/- | D/D/-/- | 1/1/-/- |
| 109 | 102 | 0/0/0/-/- | B2/C/-/- | 1A/1B/-/- |
| 110 | 103 | 0/-/-/-/- | -/-/-/- | -/-/-/- |
| 111 | 104 | 0/0/0/-/- | D/D/-/- | 1A/1A/-/- |
| 112 | 105 | 0/0/0/-/- | B3/B3/-/- | 3/3/-/- |
| 113 | 106 | 0/0/-/-/- | B3/-/-/- | 3/-/-/- |
| 114 | 107 | 0/0/0/-/- | D/D/-/- | 1/1/-/- |
| 115 | 108 | 0/0/-/-/- | D/-/-/- | 1A/-/-/- |
| 116 | 109 | 0/0/-/-/- | B3/-/-/- | 3/-/-/- |
| 117 | 110a | 0/0/0/-/- | B3/B3/-/- | 3/3/-/- |
| 118 | 110b | 0/0/0/-/- | C/C/-/- | 5/5/-/- |
| 119 | 111 | 0/0/-/-/- | B3/-/-/- | 3/-/-/- |
| 120 | 112 | 0/0/0/-/- | B2/B2/-/- | 3/3/-/- |
| 121 | 113 | 0/0/0/-/- | B/B/-/- | 3/3/-/- |
| 122 | 114 | 0/0/0/-/- | C/C/-/- | 2/2/-/- |
| 123 | 115 | 0/0/0/0/- | D/D/D/- | 1/1/1/- |
| 124 | 116 | 0/0/0/c.f./- | D/D/c.f./- | 1A/1A/c.f./- |
| 125 | 117 | 0/0/0/0/- | D/D/D/- | 1A/1A/1A/- |
| 126 | 118 | 0/0/0/0/- | D/D/D/- | 1A/1A/1A/- |
| 127 | 119 | 0/0/0/-/- | B/B3/-/- | 3/5/-/- |
| 128 | 120 | 0/0/0/0/0 | D/D/D/D | 1/1/1/1 |
| 129 | 121 | 0/0/0/0/0 | A/B/D C/D | 3/2/5/5 |
| 130 | 122 | 0/0/0/c.f./- | D/D/c.f./- | 1B/1B/c.f./- |
| 131 | 123 | 0/0/-/-/- | D/-/-/- | 1/-/-/- |
| 132 | 124 | 0/0/0/0/- | D/D/D/- | 1B/1B/1B/- |
| 133 | 125 | 0/0/0/0/0 | C/C/C/C | 2/2/5/5 |
| 134 | 126 | 0/0/-/-/- | D/-/-/- | 1A/-/-/- |
| 135 | 127 | 0/0/0/0/0 | A/A/A/A | 3/3/3/3 |
| 136 | 128 | 0/0/0/c.f./c.f. | D/D/c.f./c.f. | 1/1/c.f./c.f. |
| 137 | 129 | 0/0/0/-/- | D/D/-/- | 1B/1B/-/- |
| 138 | 130 | 0/0/0/-/- | D/D/-/- | 1C/1C/-/- |
| 139 | 131 | 0/0/-/-/- | B/-/-/- | 3/-/-/- |
| 140 | 132 | 0/0/0/0/- | D/D/D/- | 1/1/1/- |
| 141 | 133 | 0/0/0/-/- | D/D/-/- | 1/1/-/- |
| 142 | 134 | 0/0/0/-/- | D/D/-/- | 1/1/-/- |
| 143 | 135a | 0/0/0/-/- | D/D/-/- | 1/1/-/- |
| 144 | 135b | 0/0/0/-/- | D/D/-/- | 1B/1B/-/- |
| 145 | 136 | 0/0/0/-/- | D/D/-/- | 1/1/-/- |
| 146 | 137 | 0/0/0/-/- | D/D/-/- | 1/1/-/- |
| 147 | 138 | 0/0/0/0/- | D/D/D/- | 1A/1A/1A/- |
| 148 | 139 | 1/1/-/-/- | A/-/-/- | 3/-/-/- |
| 149 | 140 | 0/0/0/0/0 | C/C/D/D | 3/3/1C/1C |
| 150 | 141 | 0/0/0/-/- | B/D/-/- | 3/1B/-/- |
| 151 | 142 | 0/0/-/-/- | D/-/-/- | 1A/-/-/- |
| 152 | 143 | 0/0/-/-/- | C3/-/-/- | 5/-/-/- |
| 153 | 144 | 0/0/0/0/- | D/D/D1/- | 1A/1A/1A/- |
| 154 | 145 | 2/2/2/c.f./- | D1/D/c.f./- | 1A/1A/c.f./- |
| 155 | 146 | 0/0/0/0/- | D/D/D/- | 1A/1A/1A/- |
| 156 | 147 | 0/0/0/0/- | B/D1/D/- | 3/1/1/- |
| 157 | 148 | 0/0/0/0/- | D/D1/D/- | 1C /1C/1C/- |
| 158 | 149 | 0/4/4/-/- | D/D/-/- | 1A/1A/-/- |
| 159 | 150 | 0/0/0/0/- | B/B/B/- | 3/3/3/- |

**Note**:

OKM = O’Kelly-Marotta classification of degree of aneurysm filling: A = total filling; C = entry remnant; D = not filling. Degree of stasis: prolongation of stasis into 1 = arterial; 2 = capillary; 3 = venous phase

CSC = Cekirge-Saatci classification of degree of aneurysm filling: Class 1 = complete occlusion, 1A: with full patency o the integrated branch, 1B: with the branch reduced in caliber, 1C: with no antegrade filling of the branch, Class 2 = neck filling, Class 3 = incomplete occlusion with aneurysm filling, Class 4 = aneurysm filling – this class is reserved for an immediate postoperative result based on end-of-treatment DSA, 4A: with contrast stagnation, 4B: without contrast stagnation, Class 5 = stable remodeling with flow modification, i.e. filling in the neck region, which stays unchanged or reduced in two consecutive control angiographies at least six months apart in a period of not less than one year; exceptionally one control angiography could be sufficient for definition of class 5 in selected cases of contrast filling of the branch coming off the sac, with an appearance of a different vessel course than the original, e.g. tortuous or dilated, given that it is in continuation with the parent artery with no sac filling

c.f. “carried forward”, data points that were carried forward for further analysis

A range of ± 3 months of the exact examination time point was accepted to be included into the group of 6 months, 12 months, 24 months and 36 months follow-up respectively.

**Statistics - Multivariate Analysis:**

We conducted two multivariate analyses:

Including cases at the 30-days follow-up examination, we conducted a univariate analysis to find factors associated with thrombotic-ischemic complications. Thrombus formation or periprocedural stroke occurring at treatment or within the first 30 days after treatment were defined as complication and independent variable for the analysis.

Including cases at the 6-months follow-up examination, we conducted a univariate analysis to find factors associated with aneurysm occlusion rates at 6-months follow-up. Complete or near complete occlusion OKM C and OKM D was the independent variable for the analysis.

For both analyses we extracted the following dependent variables from the individual patient data: size and neck width of the aneurysm, age, gender, anterior vs. posterior circulation, proximal vs. distal location, saccular vs. non-saccular shape of the aneurysm and whether vessel branches originate from the treated aneurysm. Distal location was defined as distal to the MCA bifurcation, distal to the A1/anterior communicating artery (Acom) complex and distal to the P1 segment of the posterior cerebral artery (PCA).

Independent variables significantly associated with treatment complications in the first analysis (or with aneurysm occlusion in the second analysis) in the univariate analysis were analyzed in a binary logistic regression. For the multivariate analysis the results of the regression model were calculated by the Wald test with a stepwise variable selection, stopping criterion p<0.1 and reinclusion criterion p<0.05, and expressed using p-values and related Odds Ratio. Statistical analyses were performed with SPSS Version 24 (IBM, Armonk, New York).
